# Novel vs established cryoballoon ablation system for atrial fibrillation: A systematic review and meta-analysis

**DOI:** 10.1016/j.hroo.2024.10.022

**Published:** 2024-11-07

**Authors:** Jeanne du Fay de Lavallaz, Sven Knecht, Tobias Reichlin, Philipp Krisai, Diego Mannhart, Teodor Serban, Laurent Roten, Rebecca Arnet, Corinne Isenegger, Judith Minder, Fabian Jordan, Christian Sticherling, Michael Kühne, Patrick Badertscher

**Affiliations:** 1 Department of Cardiology, University Hospital Basel, Basel, Switzerland; 2 Department of Cardiology, Cardiovascular Research Institute Basel, Basel, Switzerland; 3 Department of Cardiology, Inselspital Bern, University of Bern, Bern, Switzerland

**Keywords:** Atrial fibrillation, Pulmonary vein isolation, Cryoballoon ablation, POLARx, Catheter ablation

## Abstract

**Background:**

Recently, a novel cryoballoon ablation system (POLARx) for the treatment of atrial fibrillation has been introduced.

**Objective:**

We aimed at systematically reviewing the efficacy and safety of the POLARx compared with the ArcticFront system.

**Methods:**

Structured systematic database search for articles published between 2021 and 2024 reporting the efficacy and/or safety of the POLARx system for atrial fibrillation ablation. The co-primary endpoints were the long-term efficacy and safety of the POLARx system.

**Results:**

Of the 24 studies with 5364 patients (weighted mean age 62.4 years) included, 15 compared the POLARx system (1746 patients) with the ArcticFront system (2282 patients). Despite significantly lower temperatures at isolation (POLARx –46.3 °C, ArcticFront –31.6 °C, *P* < .01) and nadir temperatures (POLARx –56.5 °C, ArcticFront –47.8 °C, *P* < .01), the POLARx system did not show a better acute (98.9% and 99.2% successfully ablated patients and 99.5% and 99.8% successfully ablated pulmonary veins in the POLARx and ArcticFront groups, respectively) or long-term (after a weighted mean follow-up of 12.6 months, the success rate was 69.5% with POLARx and 60.2% with ArcticFront, *P* = .98) efficacy. While most complications were similar between the POLARx and ArcticFront groups, the incidence of phrenic nerve palsies in the pooled cohorts (all POLARx vs all ArcticFront control patients) differed (2.7% vs 1.6% in the POLARx vs ArcticFront groups; odds ratio 1.79, 95% confidence interval 1.14–2.83, *P* = .01).

**Conclusion:**

The novel POLARx system provided similar efficiency and acute/long-term efficacy but showed a higher incidence of phrenic nerve palsies compared with the ArcticFront system.

## Graphical abstract

**Figure:**
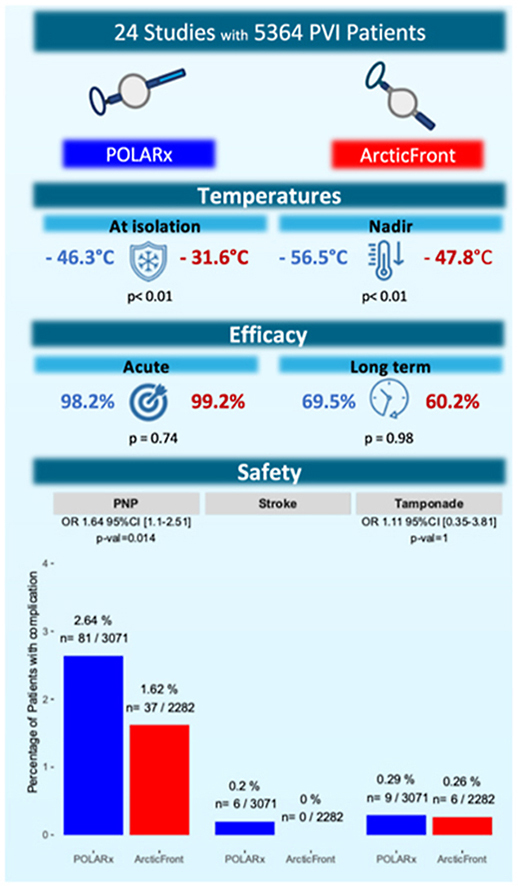

**Key Findings**

▪ In this systematic review and meta-analysis comparing the POLARx and ArcticFront cryoballoon ablation systems, we found similar acute efficacy, with high rates of successful ablation (98.2% for POLARx and 99.9% for ArcticFront) and comparable long-term outcomes.
▪ The POLARx system had a higher incidence of phrenic nerve palsy (PNP) compared with ArcticFront (2.7% vs 1.6%), with a 79% increased odds of experiencing PNP in the POLARx group.
▪ There is a need for further studies, particularly on the newer POLARx FIT system, to assess its impact on PNP incidence and to clarify the mechanisms behind the safety differences observed.

## Introduction

Cryoballoon ablation (CBA) has been proven to be as safe and efficient compared with radiofrequency energy for the treatment of atrial fibrillation (AF).^1^^,^^2^ Until recently, the ArcticFront CBA system (Medtronic) has been the only available CBA system and is currently available in its fourth generation.^3^

The POLARx cryoablation system (Boston Scientific) was introduced in May 2020, and several observational studies reported their initial experience with this novel CBA system. A first randomized controlled trial (RCT) was recently published.^4^ Despite lower measured balloon nadir temperatures, POLARx had a similar procedural efficacy and safety in comparison with the ArcticFront CBA system.^5^, ^6^, ^7^, ^8^, ^9^, ^10^, ^11^, ^12^, ^13^, ^14^, ^15^, ^16^, ^17^, ^18^

Differences in design and cooling properties of the 2 CBA systems could potentially lead to subtle but clinically relevant differences in efficacy and safety. Given the relative rarity of complications observed during pulmonary vein isolation, individual studies were underpowered to adequately assess a potential clinically relevant safety difference between the 2 CBA systems.

Hence, we performed a systematic review and meta-analysis to provide an up-to-date comparison of the efficacy and safety of a novel CBA system, the POLARx ablation system, vs the current standard of care, the ArcticFront CBA system.

### Objectives

The aim of the present study was to systematically review the current literature on the efficacy and safety of the novel POLARx CBA system in comparison with the current standard of care, the second- or fourth-generation ArcticFront CBA ablation system, in an unselected population of patients undergoing AF ablation and provide a meta-analytic summary on available endpoints.

## Methods

The results are presented according to the PRISMA (Preferred Reporting Items for Systematic Reviews and Meta-Analysis) statement^19^ on systematic reviews (Supplemental Table 1).

### Data sources and search

The search strategy was designed with the guidance of a research librarian. A first database search in PubMed, MEDLINE, and Embase was performed on October 12, 2023, by combining synonyms of the terms “atrial fibrillation,” “cryoballoon,” and “POLARx” (Supplemental Appendix) and included abstracts published from inception to that current date. The search was conducted a second time on April 1, 2024, to include the most recent studies.

### Study selection

Abstracts and studies were included if they followed prespecified criteria: (1) the study assessed the efficacy and/or safety of the POLARx CBA system for pulmonary vein isolation, (2) was not a meta-analysis or review, and (3) did not focus on a highly selected population. Studies were included independently of the presence of a control group (other CBA system, other ablation energy), but all pooled or meta-analytic comparisons were predefined to be calculated with the ArcticFront group only (no inclusion of other energy sources or ablation catheters). Congress abstracts were excluded from analyses. We did not differentiate between study assessing exclusively first time or also redo ablations.

The selection, validation, and data extraction of the study were carried out by 4 independent researchers from the study team (R.A., C.I., D.M., J.M.) in a dedicated REDCap (Research Electronic Data Capture) database hosted at the University Hospital Basel. A fifth researcher (J.d.F.d.L.) validated the co-primary endpoints in a blinded manner. More information on the selected studies and the extracted data is presented in the Supplemental Appendix.

All selected studies were again reviewed together by 2 independent researchers (J.d.F.d.L. and J.M.) to exclude multiple publications based on the same cohort. This was achieved by comparing publications stemming from similar authors and affiliations. When several publications assessed the same cohort, the latest publication with the longest follow-up was selected. Before exclusion of the studies published earlier by the same authors, safety and procedural data were assessed between publications to ensure their presence in the most recent paper.

### Endpoints

The co-primary endpoints were the long-term efficacy and the safety of the POLARx CBA system vs ArcticFront system. As no exact definition of the “long-term” efficacy of cryoballoon ablation is accepted throughout the literature, we defined long-term efficacy in the current analysis as the freedom from recurrence of AF at least 6 months postablation. In general, only events reported after the blanking period were considered. The safety was defined as any complication reported until the patient’s discharge following the ablation. Complications were defined as any phrenic nerve palsy (PNP) and/or (1) those the authors explicitly mentioned in their methods or results or (2) "any" associated with the intervention in their Methods or Results. Secondary endpoints were the acute procedural efficacy and procedural outcomes (procedural time, ablation time, time to isolation, temperature at isolation, nadir temperature, and fluoroscopy time).

### Evaluation of study quality

The quality of the included studies was assessed using the Newcastle-Ottawa scale^20^ quality criteria for observational studies and the RoB 2 tool^21^ for RCTs. Minimal criteria for observational studies to belong to the current analysis were clear ascertainment of exposure and outcome and transparent cohort/case definition, and for case-control studies, clear control description. These criteria belonged to the study selection process; hence, no studies were excluded in a post hoc manner given their poor quality.

### Statistical analysis

All analyses were performed using the R statistical program (version 4.1.2; R Foundation for Statistical Computing) by following the Cochrane Collaboration recommendations.^22^ The results are reported according to the PRISMA statement^19^ and the most recent guidelines.

All baseline characteristics of the patients were recorded as mean with SD or as median and interquartile range. The median and interquartile range were then converted into mean and SD to allow quantitative summaries as proposed in previous research.^23^

Overall patients’ characteristics are presented as weighted mean or weighted percentages and compared using a weighted univariable linear regression.

Meta-analytic summaries were provided for primary and secondary endpoints using the adequate function of the meta R package^24^: metagen to assess standardized mean difference (SMD) for continuous outcomes when a comparison between the POLARx CBA system and the ArcticFront CBA system was performed, metagen to assess weighted mean difference for continuous outcomes when the POLARx system was assessed alone, metabin to assess the risk difference (RD) for binary outcomes when a comparison between the POLARx and the ArcticFront CBA system was performed, and metabin to assess logit-transformed proportions (plogit) for binary outcomes when the POLARx system was assessed alone.

Results are graphically presented in forest plots. To estimate the variability between studies, the risk estimates, and the confidence intervals (CIs), random-effects models by inverse variance method were used, as proposed by the meta R package,^24^ which accounts for intra- and interstudy variance. The variance between studies was calculated using the DerSimonian-Laird estimator, and the CI of the τ^2^ was calculated by using the Jackson method. Statistical heterogeneity was evaluated with the I^2^ statistic, and an I^2^ >50% indicates a high level of heterogeneity.

Meta-regression was used to correct for the publication year when assessing the incidence of PNP.

For the safety analysis and given the relative rarity of events, patients from the studies reporting a specific complication were pooled depending on the ablation system used (POLARx or ArcticFront CBA system) and a 1-step percentage was calculated in different groups: (1) the overall pooled population from all selected studies (the population from all studies assessing the safety of POLARx—with or without ArcticFront CBA system control group—vs the population ablated with the ArcticFront system), (2) the pooled population from the studies assessing POLARx vs a dedicated ArcticFront CBA system as control group, and (3) the pooled population from all the studies assessing POLARx vs a fourth-generation ArcticFront CB.

A *P* value ≤.05 was considered statistically significant.

## Results

### Selected studies

Of the 371 abstracts screened, 275 were excluded for not focusing on the evaluation of the POLARx CBA system and 25 were excluded as they were meta-analyses or reviews. During a full-text review of the 72 remaining studies, 33 were excluded, as they evaluated the same cohort several times. Furthermore, 4 publications were excluded, as they did not record any of the outcome of interest, and 11 studies were excluded, as these were only available as abstracts.

Eventually, 24 studies evaluating independent cohorts were selected for analyses^4^^,^^6^, ^7^, ^8^, ^9^, ^10^, ^11^, ^12^, ^13^, ^14^, ^15^, ^16^, ^17^^,^^25^, ^26^, ^27^, ^28^, ^29^, ^30^, ^31^, ^32^, ^33^, ^34^, ^35^ (Supplemental Figure 1), investigating a total of 5364 patients (3082 ablated with POLARx, 2282 ablated with ArcticFront CBA system).

Over the 24 included studies, 15^4^^,^^6^, ^7^, ^8^, ^9^, ^10^, ^11^^,^^13^, ^14^, ^15^, ^16^, ^17^^,^^26^^,^^31^^,^^33^compared the POLARx with the ArcticFront CBA system (for a total of 1746 patients ablated with the POLARx system), while 8 focused only on POLARx and 1 compared POLARx with a pulsed field ablation systemCliquez ou appuyez ici pour entrer du texte. (this study was assessed without its control group throughout the analysis).^28^ Among the studies comparing POLARx with the ArcticFront CBA system (n = 15 of 24), 2 compared POLARx with the second-generation ArcticFront CB^26^^,^^31^ and 13 compared POLARx with the fourth-generation ArcticFront CB.^4^^,^^6^, ^7^, ^8^, ^9^, ^10^, ^11^, ^12^, ^13^, ^14^, ^15^, ^16^, ^17^ Four^10^^,^^16^^,^^17^^,^^28^ studies were retrospective, 20 were prospective, 10 were monocentric, and 14 were multicentric. Twenty-three studies were observational, and 1 RCT^4^ was recorded (POLARx vs the fourth-generation ArcticFront CB). Studies were published between 2021 and 2024 (Supplemental Table 2). Twenty-two studies investigated acute or long-term efficacy outcomes (22 studies reported acute efficacy per patient, 15 reported acute efficacy per vein, 15 reported what they defined as being long-term efficacy outcomes, of which 13 had at least 6 months follow-up), 22 investigated safety outcomes, and 23 investigated procedural outcomes.

### Patient characteristics

There was no difference in the weighted mean age, weighted mean percentage of enrolled women, and weighted percentage of comorbidities between the POLARx and ArcticFront groups (Table 1). Patients presented with a weighted mean age of 62.4 years (range 53 to 69 years). and the studies enrolled a weighted mean percentage of 34.9% women (range 0% to 48%). Detailed patient characteristics per group and per study are detailed in Supplemental Table 3.

**Table 1:** Weighted patients characteristic across all studies (17 comparing POLARx with an ArcticFront control system, 7 focusing on POLARx only)

Characteristic	POLARx	ArcticFront	*P* value from weighted univariable regression
Weighted mean percentage of women, %	36.9	32.2	0.57
Weighted mean age, y	62.5	62.2	0.65
Weighted mean BMI, kg/m^2^	28.3	27.8	0.24
Weighted mean percentage of stroke, %	4.7	3.7	0.62
Weighted mean percentage of hypertension, %	45.2	48.1	0.06
Weighted mean percentage of diabetes, %	8.8	10.4	0.07
Weighted mean percentage of CHF, %	10.9	9.3	0.24
Weighted mean percentage of paroxysmal AF, %	75.4	67.9	0.38
Weighted mean percentage of BBs, %	54.2	65.7	0.13
Weighted mean LA size, cm	34.6	41.5	0.28
Weighted mean LAVI, mL/m^2^	37.4	38.8	0.61
Weighted mean LVEF, %	57.7	55.7	0.11

*P* values were obtained using weighted linear regression.

AF = atrial fibrillation; BB = beta-blocker, BMI = body mass index; CHF = congestive heart failure; LA = left atrium; LAVI = left atrial volume index; LVEF = left ventricular ejection fraction.

### Acute efficacy

**Figure 1:**
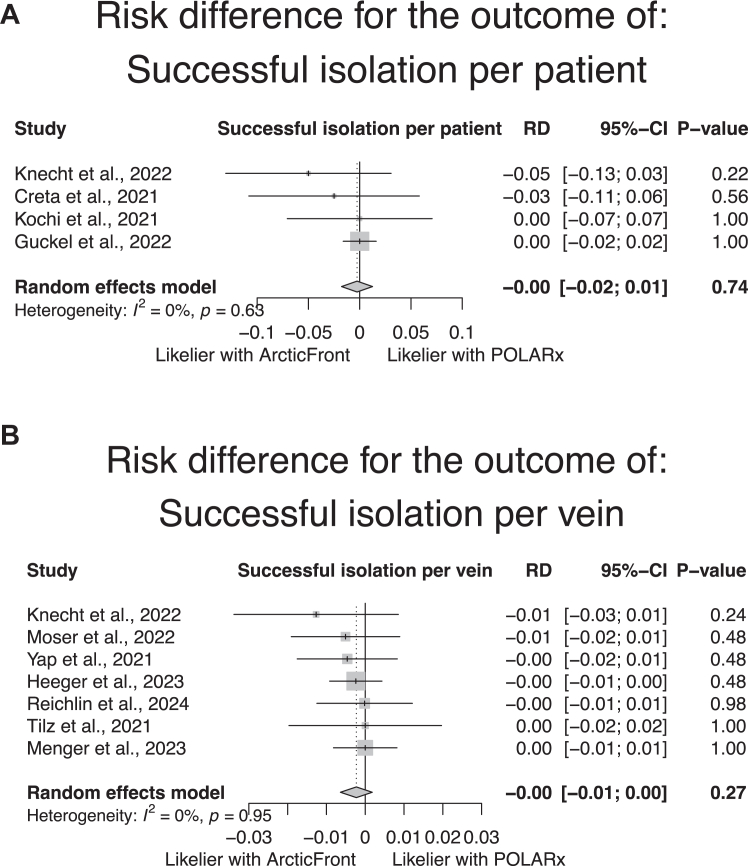
Meta-analytic summary of the acute ablation success per patient (A) and per overall number of veins (B) in the studies reporting this outcome in both the POLARx and ArcticFront control group. CI = confidence interval; RD = risk difference.

There was no difference in the weighted mean of successfully ablated patients (4 studies, POLARx 98.2% [range 95% to 100%], ArcticFront group 99.9% [range 97.5% to 100%], *P* from univariable linear regression = .38; RD 0, 95% CI –0.02 to 0.1, *P* = .74) (Figure 1A) or veins (7 studies, POLARx 99.5% [range 63.5% to 100%], ArcticFront group 99.8% [range 99.2% to 100%], *P* value from univariable linear regression = .49; RD 0, 95% CI –0.01 to 0, *P* = .27) (Figure 1B). Details regarding acute efficacy endpoints are detailed in Supplemental Table 4. Studies assessing the POLARx system again displayed a significant heterogeneity in these 2 measures (Supplemental Figures 2A and 2B).

### Long-term efficacy

**Figure 2:**
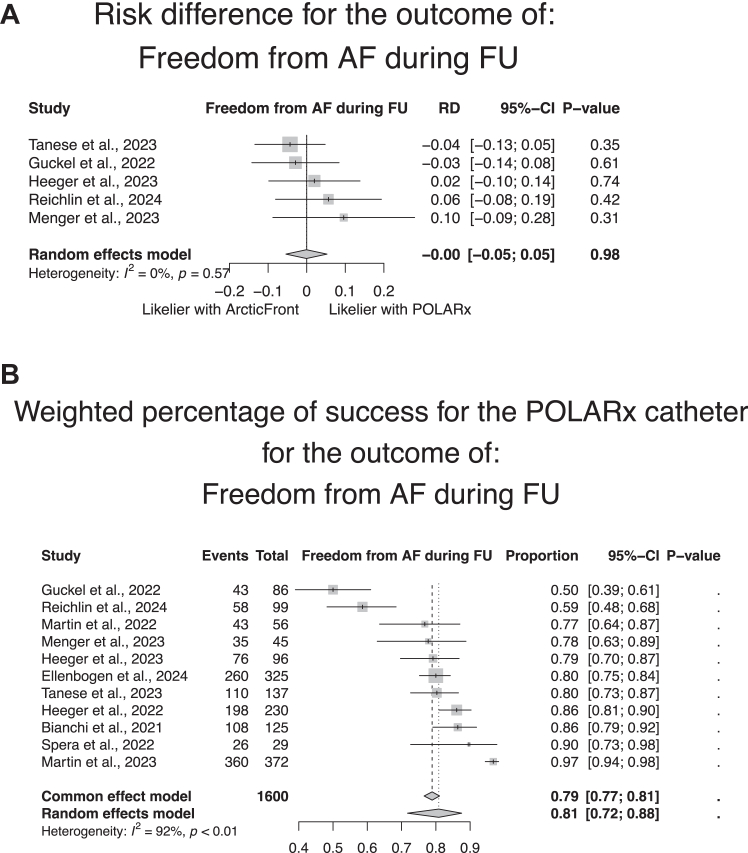
Meta-analytic summary of the long-term ablation success (≥6 months follow-up) per patient in (A) the studies reporting this outcome both in the POLARx and ArcticFront control group (A) and in the studies reporting this outcome for a POLARx cohort (B). The events correspond to the number of patients considered as free from atrial fibrillation (AF) at follow-up (FU). CI = confidence interval; RD = risk difference.

Of the 12 studies reporting long-term efficacy endpoint per patient after at least 6 months, only 5 reported a comparison between the POLARx and ArcticFront after a weighted follow-up of 12.6 months, showing no statistically significant difference between both groups (POLARx 69.5%, ArcticFront group 60.2%, *P* value from univariable linear regression = .66; RD 0.0, 95% CI –0.05 to 0.05, *P* = .98) (Figure 2A). A total of 9 studies assessed the long-term per-patient efficacy of POLARx alone and showed a significant heterogeneity in long-term efficacy (Figure 2B).

### Safety

**Figure 3:**
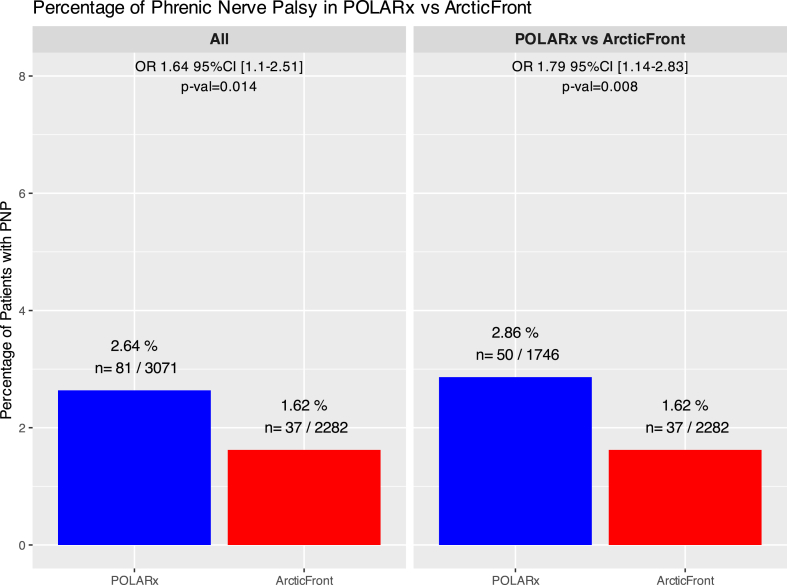
Incidence of phrenic nerve palsies (PNPs) in all studies assessing the POLARx (including the ones without a control cohort) vs all studies assessing an ArcticFront control cohort (left) and all studies assessing the POLARx together with an ArcticFront control cohort (right). The odds ratio (OR) of experiencing a PNP when POLARx was used in comparison with the control ablation system are provided with 95% confidence intervals (CIs). The *P* value provided in the graph was calculated using a Fisher test. CB = cryoballoon.

All adverse events reported by each study are presented in Supplemental Table 5. At the exception of PNP, the occurrence of complications was similar between the 2 systems (Table 2). When the overall population from all selected studies was analyzed together, a significantly higher rate of PNP (POLARx 2.7% vs ArcticFront group 1.6%; OR 1.79, 95% CI 1.14 to 2.83, *P* = .01) was reported in the POLARx pooled cohort. Similarly, when the comparison was performed in the pooled population from the studies comparing the safety of the POLARx with the ArcticFront, a similar statistically significant higher incidence of PNP was observed with POLARx (Figure 3). The incidence of PNP appeared to be moderately heterogeneous between studies (Supplemental Figure 3).

**Table 2:** Number of patients presenting with an adverse event in the pooled cohorts of patients undergoing pulmonary vein isolation with the POLARx or ArcticFront group system

Type of complication	POLARx group	ArcticFront group
Phrenic nerve palsy	2.64 (81/3071)	1.62 (37/2282)
Air embolism	0.38 (11/2858)	0 (0/2066)
Stroke	0.2 (6/3071)	0 (0/2282)
TIA	0.07 (2/2942)	0 (0/2180)
Cardiac tamponade	0.29 (9/3071)	0.26 (6/2282)
Hematoma	0.47 (12/2570)	0.41 (9/2180)
Hemoptysis	0 (0/2456)	0.05 (1/2066)
Pericardial effusion	0.2 (6/2972)	0.05 (1/2180)
Third-degree AV block likely due to the intervention	0.04 (1/2828)	0 (0/2066)
Sinus arrest	0 (0/2828)	0.05 (1/2066)
Aneurysma spurium	0.07 (2/2828)	0.1 (2/2066)
Myocardial infarction	0.07 (2/2972)	0 (0/2180)
Gastroparesis	0.18 (5/2828)	0 (0/2066)
Pericarditis	0.08 (2/2456)	0 (0/2066)
Pulmonary edema	0.04 (1/2456)	0 (0/2066)

Values are % (n/n). All studies have been merged in a 1-step analysis.

AV = atrioventricular; TIA = transient ischemic attack.

Both for the POLARx as for the ArcticFront group, the majority of these PNP were reported as “transient” (POLARx 53 of 81 [65.4%] and ArcticFront 25 of 37 [67.6%]). The 10^6^^,^^9^^,^^11^^,^^16^^,^^17^^,^^26^^,^^27^^,^^29^^,^^33^^,^^34^ studies reporting the 26 patients with a “persistent” PNP (PolarX 23 of 81, ArcticFront 12 of 37) followed up the persistence of this complication up to different time points (Supplemental Table 6), with only a minority of studies following their patients up to 12 months.^6^^,^^9^^,^^16^^,^^26^

### Interventional details

**Figure 4:**
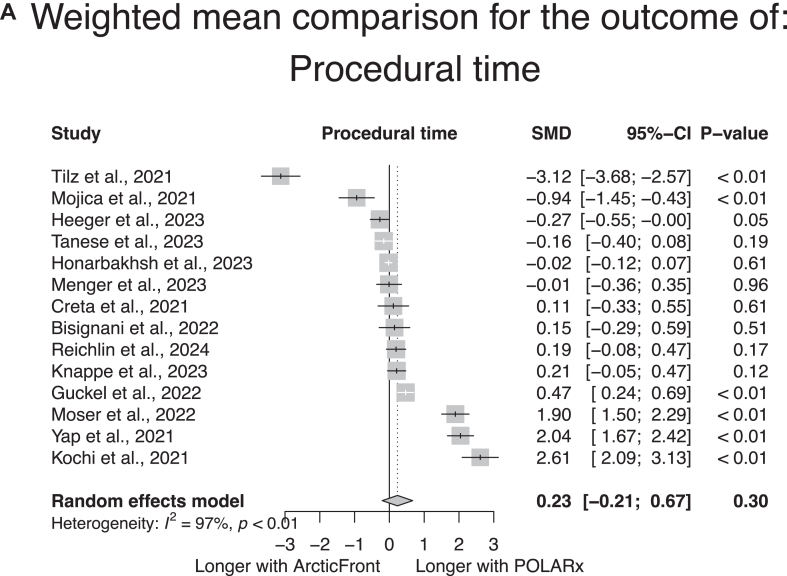

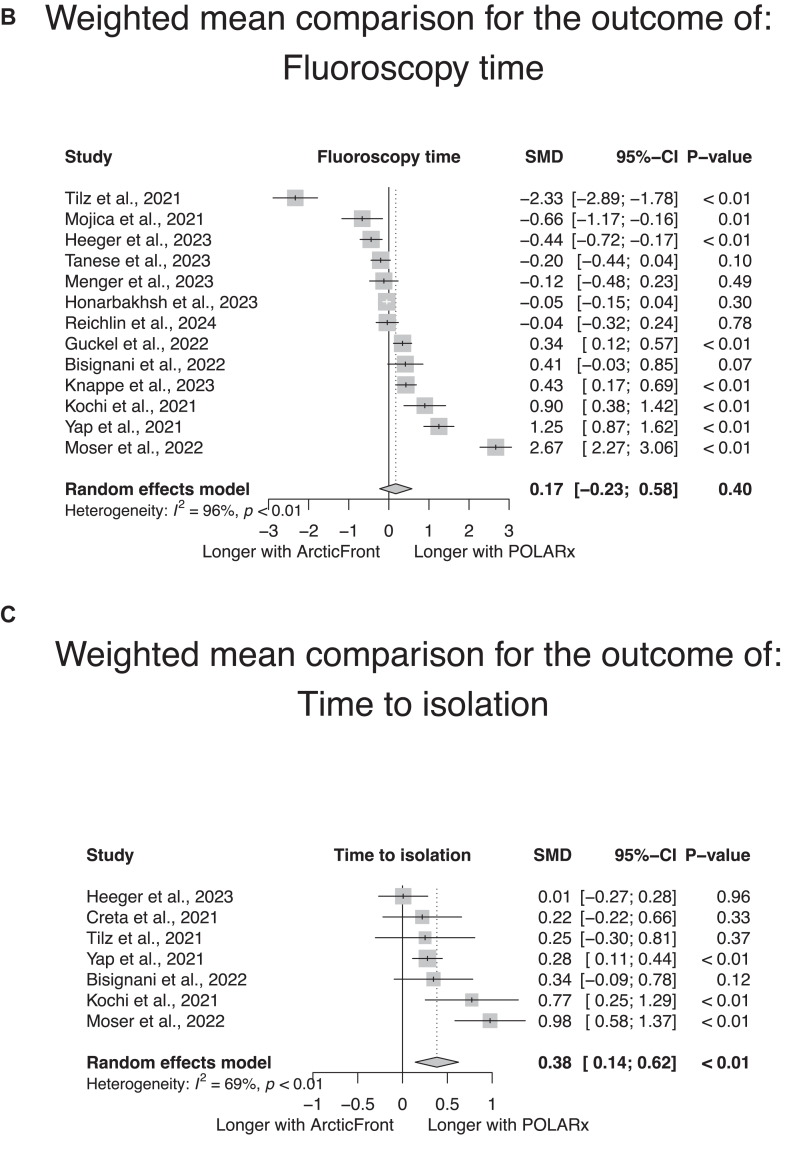
Meta-analytic summary of the procedural time (A), fluoroscopy time (B), and mean time to isolation (C) in the studies reporting this outcome both in the POLARx and ArcticFront control groups. CI = confidence interval; SMD = standardized mean difference.

**Figure 5:**
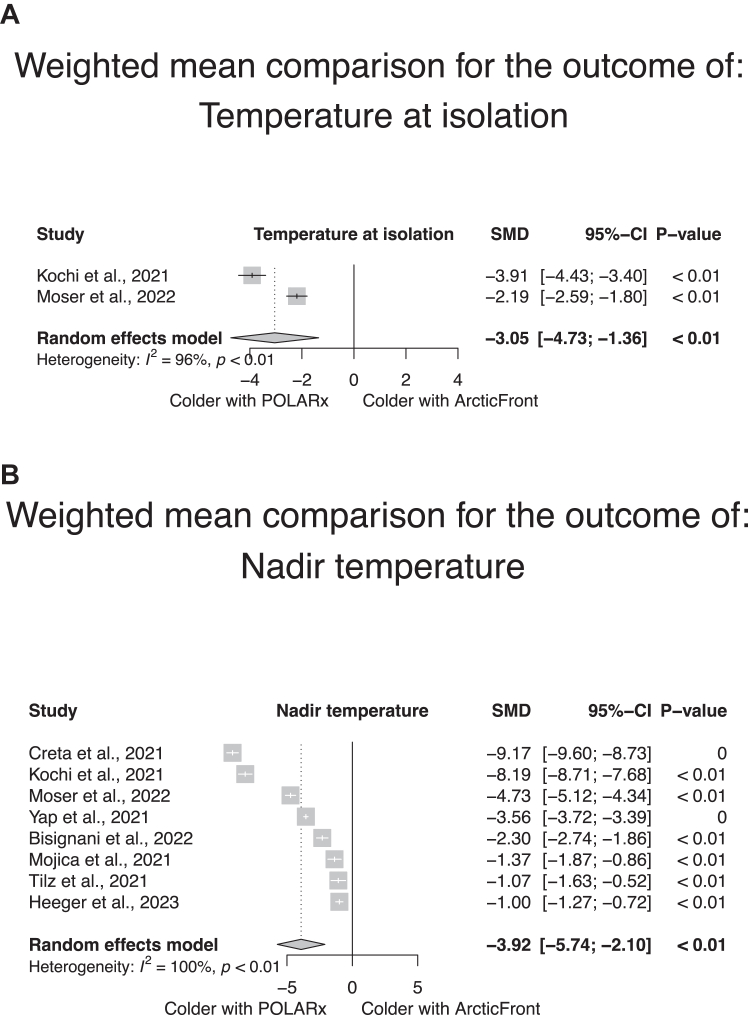
Meta-analytic summary of the mean isolation temperature (A) and mean nadir temperature (B) in the studies reporting this outcome both in the POLARx and ArcticFront control group. CI = confidence interval; SMD = standardized mean difference.

Interventional details per study and per group are given in Supplemental Table 7. As shown in Figure 4, there was no significant difference in the procedural time (14 studies, weighted mean in the POLARx group 79.2 minutes [range 38 to 177 minutes], in the ArcticFront group 80.9 minutes [range 38 to 101 minutes]; SMD according to a random-effect model 0.23, 95% CI –0.21 to 0.67, *P* = .30) (Figure4A) or fluoroscopy duration (13 studies, weighted mean in the POLARx group 15 minutes [range 8.5 to 30 minutes], in the ArcticFront group 13.5 minutes [range 8 to 24 minutes]; SMD according to a random-effect model 0.17, 95% CI –0.23 to 0.58, *P* = .40) (Figure 4B) between both systems. The POLARx system was associated with longer mean time to isolation (7 studies, weighted mean in the POLARx group 45.4 seconds [range 17 to 59 seconds], weighted mean in the ArcticFront group 40.1 seconds [range 16 to 42 seconds]; SMD according to a random-effect model 0.38, 95% CI 0.14 to 0.62, *P <* .01) (Figure 4C). Studies assessing the POLARx system displayed a significant heterogeneity in these 3 durations, with mean procedural time ranging from 38 to 177 minutes, mean fluoroscopy duration ranging from 8.5 to 30 minutes, and mean time to isolation ranging from 17 to 59 seconds (Supplementary Figures 4A–4C). Given the low number of studies reporting the net ablation time, no quantitative summary of this measure was attempted. As shown in Figure 5, the POLARx system showed lower temperatures at isolation (3 studies, weighted mean in the POLARx group –46.3 °C [range –47 °C to –38 °C], in the ArcticFront group –31.6 °C [range –32 °C to –30 °C]; SMD according to a random-effect model –3.1 °C, 95% CI –4.7 °C to –1.4 °C, *P* < .01) (Figure 5A) and colder nadir temperatures (8 studies, weighted mean in the POLARx group –56.5 °C [range –57 °C to –47 °C], in the ArcticFront group –47.8 °C [range –50 °C to –47 °C]; SMD according to a random-effect model –3.9 °C, 95% CI –5.7 to –2.1, *P* < .01) (Figure 5B) than the ArcticFront control system. There was again a high heterogeneity in the temperatures measured with the POLARx system among studies (Supplementary Figures 5A and 5B).

### Assessment of study quality

The quality of observational studies evaluated by the Newcastle-Ottawa scale and of the only available RCT using the RoB 2 tool is presented in Supplemental Figure 6. On average, the included observational studies were of moderate to good quality and the RCT^4^ showed a low risk of bias.

## Discussion

The aim of this systematic review and meta-analysis was to provide an up-to-date comparison of the efficacy and safety of a novel CBA system, the POLARx ablation system, compared with the ArcticFront CBA system.

We report the following major findings. First, there was no difference in acute efficacy on a per-patient or per-vein basis, with 98.2% and 99.9% successfully ablated patients and with 99.5% and 99.8% successfully ablated PVs in the POLARx and ArcticFront groups, respectively. Second, there was no statistically significant difference regarding long-term efficacy. Third, we found a significant difference in safety regarding the most feared complication of CBA for AF. The incidence of PNP was higher for the POLARx CBA system compared with the ArcticFront CBA system (2.7% vs 1.6%). The odds of experiencing PNP were 79% higher in the POLARx group. Fourth, procedural characteristics such as procedure duration or fluoroscopic times were similar between both CBA systems. The POLARx system showed significantly colder nadir temperatures and temperatures at isolation but with a longer time to isolation.

While the present analysis did not highlight any differences in efficiency and efficacy of both CBA systems, the observed difference in safety is noteworthy. Because randomized evidence is limited to a single trial^4^ and the observational studies^6^, ^7^, ^8^, ^9^, ^10^, ^11^, ^12^, ^13^, ^14^, ^15^, ^16^, ^17^^,^^25^, ^26^, ^27^, ^28^, ^29^, ^30^, ^31^, ^32^, ^33^, ^34^, ^35^ were of small or moderate size, an accurate assessment of a possible higher PNP incidence in the individual studies proved difficult given the relative rarity of this complication. When pooling all reports on safety of POLARx compared with second- or fourth-generation ArcticFront CB^4^^,^^6^, ^7^, ^8^, ^9^, ^10^, ^11^^,^^13^, ^14^, ^15^, ^16^, ^17^^,^^26^^,^^31^^,^^33^ as performed in the current report, a statistically significant higher incidence of PNP became evident. Given the large sample size achieved by our meta-analysis, a type I error is unlikely. The higher incidence of PNP could result from the lower nadir temperatures consistently achieved by the POLARx CBA system, as reported in the current work. However, the lower nadir temperatures internally measured with the POLARx CBA system do not necessarily translate to a lower balloon surface temperature and lower temperatures at the balloon-tissue interface.^3^^,^^31^ The fact that time to effect was found to be longer with POLARx despite lower nadir temperatures suggests other causes for the higher incidence of PNP with the POLARx CBA system. Small differences in the catheter design between the 2 CBA systems might cause the observed differences in measured internal nadir temperatures such as the position of the thermocouple, the position and injection orientation of the N_2_O injection coil, the differences in N_2_O flow, and the efficacy of the energy transfer via the used balloon material and/or balloon-tissue contact area. Two recent studies using esophageal temperature probes confirmed similar minimal esophageal temperatures during freezing between both CBA systems.^11^^,^^33^ It is conceivable that the higher incidence of PNP could be attributed to the more compliant characteristics of the POLARx CBA system (due to the constant inner balloon pressure during the inflation and freezing phase), which potentially allows the POLARx CB to be positioned more distal in the PVs when compared with the ArcticFront CB. Further studies assessing the PV depth of both CBA systems are warranted to confirm this hypothesis.

### Limitations

Several limitations of this meta-analysis should be mentioned. First, while studies reported whether the PNP were transient or persistent, only limited long-term follow-up was available for the PNP considered as persistent. Prior studies using the ArcticFront CBA system showed that 97% of PNP recovered within 12 months.^18^ It is likely that PNP recovery will also occur in most patients using POLARx, Second, there are currently only limited studies available for the newest generation of the POLARx CBA system, the POLARx FIT CBA system, and the present report does not include any patients treated with the POLARx FIT. This novel CBA system allows expansion of the balloon diameter from 28 to 31 mm.^36^ Theoretically, a larger balloon should provide more proximal occlusion and increase the distance to the phrenic nerve when ablating the right superior PV. Further studies assessing the incidence of PNP using the POLARx FIT CBA system are needed. Third, for some outcome parameters, there was significant heterogeneity between studies, likely reflecting variations in study design, slight in outcomes definition and reporting (eg, no denomination into AF, atrial flutter, or atrial tachycardia in some studies), varying follow-up duration (impacting on long-term outcomes), and low incidence of events (rare complications). In an attempt to mitigate this high heterogeneity, we consistently used random-effect models, but we could not correct for underlying study-specific heterogeneity sources (such as the observational character of the largest majority of studies, center-specific procedural differences, and varying follow-up lengths). Eventually, we found a limited number of studies reporting truly long-term outcomes (≥12 months), so the efficacy past this time point cannot be accurately evaluated in the present analysis.

## Conclusion

In conclusion, the POLARx CBA system provided similar efficiency and efficacy but a higher incidence of PNP compared with the ArcticFront CBA system. Small technical details in catheter design are most likely responsible for the observed safety difference.

## Disclosures

Jeanne du Fay de Lavallaz has received research funding from the University of Basel and from the Swiss Heart Foundation. Teodor Serban has received research funding from the Swiss Academy of Medical Sciences and the Gottfried and Julia Bangerter-Rhyner Foundation. Sven Knecht has received funding from the 'Stiftung für Herzschrittmacher und Elektrophysiologie. Tobias Reichlin has received research grants from the Swiss National Science Foundation, the Swiss Heart Foundation, the sitem-insel Support Funds, Biotronik, Boston Scientific, and Medtronic, all for work outside the submitted study; received speaker/consulting honoraria or travel support from Abbott/SJM, Bayer, Biosense Webster, Biotronik, Boston Scientific, Farapulse, Medtronic, and Pfizer-BMS, all for work outside the submitted study; and received support for his institution’s fellowship program from Abbott/SJM, Biosense Webster, Biotronik, Boston Scientific, and Medtronic for work outside the submitted study. Michael Kühne has received reports grants from the Swiss National Science Foundation (nos. 33CS30_148474, 33CS30_177520, 32473B_176178), the Swiss Heart Foundation, the Foundation for Cardiovascular Research Basel and the University of Basel, Bayer, BMS, Boston Scientific, Daiichi Sankyo, and Pfizer; personal fees from Abbott, Boston Scientific, and Daiichi Sankyo; and royalties from Springer Nature, all outside the submitted work. Christian Sticherling has served on the advisory board for Medtronic Europe and Boston Scientitic Europe; received educational grants from Biosense Webster and Biotronik; received research grant support from the European Union’s FP7 program and Biosense Webster; and received lecture and consulting fees from Abbott, Medtronic, Biosense Webster, Boston Scientific, MicroPort, and Biotronik, all outside the submitted work. Patrick Badertscher has received research funding from the University of Basel, the Stiftung für Herzschrittmacher und Elektrophysiologie, the Freiwillige Akademische Gesellschaft Basel, and Johnson & Johnson, all outside of the submitted work; and personal fees from Abbott. All other authors disclose no conflicts.
