# Supplementary material for: Novel vs established cryoballoon ablation system for atrial fibrillation: A systematic review and meta-analysis
Source: Heart Rhythm O2. 2024 Nov 7;6(1):21–31. doi: 10.1016/j.hroo.2024.10.022 (PMC11993791; doi:10.1016/j.hroo.2024.10.022)
Supplement: Supplemental Material [file mmc1.docx]

**Efficacy and Safety of a Novel versus Established Cryoballoon Ablation System for Atrial fibrillation: A Meta-Analysis**

[Supplemental methods 2](#_Toc179616445)

[General search strategy 2](#_Toc179616446)

[Search strategy 2](#_Toc179616447)

[Pubmed 2](#_Toc179616448)

[Embase 2](#_Toc179616449)

[Study selection and data preparation 2](#_Toc179616450)

[Data extraction 2](#_Toc179616451)

[Supplemental tables 3](#_Toc179616452)

[Supplemental table 1 : PRISMA reporting guidelines 3](#_Toc179616453)

[Supplemental table 2 : Study characteristics 6](#_Toc179616454)

[Supplemental table 3 : Patients characteristics per study and per ablation system 8](#_Toc179616455)

[Supplemental table 4 : Acute efficacy outcomes per study and per ablation system 12](#_Toc179616456)

[Supplemental table 5 : Safety outcomes in the each study and group 15](#_Toc179616457)

[Supplemental table 6 : Latest timepoint the persistent PNP were documented 19](#_Toc179616458)

[Supplemental table 7 : Procedural details per study and per ablation system 20](#_Toc179616459)

[Supplemental figures 23](#_Toc179616460)

[Supplemental figure 1 : Flow chart 23](#_Toc179616461)

[Supplemental Figure 2 : Acute efficacy outcome 24](#_Toc179616462)

[Supplemental Figure 3 : Weighted percentage of phrenic nerve palsies using POLARx accross all studies reporting this endpoint 25](#_Toc179616463)

[Supplemental figure 4: Procedural time outcomes 26](#_Toc179616464)

[Supplemental Figure 5d : Procedural temperature outcomes 28](#_Toc179616465)

[Supplemental Figure 6 : Studies quality 29](#_Toc179616466)

# Supplemental methods

### General search strategy

To ensure completeness of the search we used both words or truncated words and MESH terms. No limitations of languages were applied. References list of each article was reviewed for potential missed studies.

## Search strategy

### Pubmed

"polar x" or "POLARx" or ("boston" and "cryoballoon" and ("atrial fibrillation" OR "AF" OR "AFIB"))

### Embase

'polar x' OR 'POLARx' OR ('boston' AND 'cryoballoon' AND ('atrial fibrillation' OR 'af' OR 'afib'))

### Study selection and data preparation

The search design was conducted with the assistance of a research librarian. All initially identified studies were exported to the Mendeley Citation Software for duplicate removal. The first step of the screening consisted of an independent review by four independent researchers from the study team (RA, CI, DM, JM) of the study title and abstract. A structured questionnaire was followed for this first selection. We excluded reviews, other meta-analyses, comments or editorials. A fifth member of the team (JDF) resolved disagreements by discussion. Review of full text and final selection of studies was performed by five independent members of the study team (RA, CI, DM, JM, JDF). Disagreements were again resolved by discussion.

Studies covering a similar population were tracked through full text analysis, mainly by comparing the first author lists, references and mentions of previous results already published. For one study initially selected^1^, direct correspondence with the first authors allowed to track overlapping cohorts.

### Data extraction

Four independent researchers from the study team (RA, CI, DM, JM) reviewed each selected study for data extraction in a dedicated RedCap database hosted at the University Hospital of Basel. A fifth member of the team (JdF) reviewed the co-primary outcomes (long-term efficacy and safety) in a blinded manner. Disagreements were solved by discussion. Extracted data included study and patients populations characteristics, details of the outcome definitions, outcomes (procedural, acute and long-term efficacy, safety) and information regarding data quality.

When central details were lacking, we attempted to gather the missing data through direct communication with the authors.

# Supplemental tables

## Supplemental table 1 : PRISMA reporting guidelines

| **Section and Topic** | **Item #** | **Checklist item** | **Location where item is reported** |
| --- | --- | --- | --- |
| **TITLE** | | |  |
| Title | 1 | Identify the report as a systematic review. | Title |
| **ABSTRACT** | | |  |
| Abstract | 2 | See the PRISMA 2020 for Abstracts checklist. | Abstract |
| **INTRODUCTION** | | |  |
| Rationale | 3 | Describe the rationale for the review in the context of existing knowledge. | Page 5-6 |
| Objectives | 4 | Provide an explicit statement of the objective(s) or question(s) the review addresses. | Page 5-6 |
| **METHODS** | | |  |
| Eligibility criteria | 5 | Specify the inclusion and exclusion criteria for the review and how studies were grouped for the syntheses. | Page 7-8 Supp |
| Information sources | 6 | Specify all databases, registers, websites, organisations, reference lists and other sources searched or consulted to identify studies. Specify the date when each source was last searched or consulted. | Page 7-8 Supp |
| Search strategy | 7 | Present the full search strategies for all databases, registers and websites, including any filters and limits used. | Supp |
| Selection process | 8 | Specify the methods used to decide whether a study met the inclusion criteria of the review, including how many reviewers screened each record and each report retrieved, whether they worked independently, and if applicable, details of automation tools used in the process. | Page 7-8 Supp |
| Data collection process | 9 | Specify the methods used to collect data from reports, including how many reviewers collected data from each report, whether they worked independently, any processes for obtaining or confirming data from study investigators, and if applicable, details of automation tools used in the process. | Page 7-8 Supp |
| Data items | 10a | List and define all outcomes for which data were sought. Specify whether all results that were compatible with each outcome domain in each study were sought (e.g. for all measures, time points, analyses), and if not, the methods used to decide which results to collect. | Page 7-8 Supp |
|  | 10b | List and define all other variables for which data were sought (e.g. participant and intervention characteristics, funding sources). Describe any assumptions made about any missing or unclear information. | Page 7-8 Supp |
| Study risk of bias assessment | 11 | Specify the methods used to assess risk of bias in the included studies, including details of the tool(s) used, how many reviewers assessed each study and whether they worked independently, and if applicable, details of automation tools used in the process. | Page 9-11, Supp |
| Effect measures | 12 | Specify for each outcome the effect measure(s) (e.g. risk ratio, mean difference) used in the synthesis or presentation of results. | Page 9-11, Supp |
| Synthesis methods | 13a | Describe the processes used to decide which studies were eligible for each synthesis (e.g. tabulating the study intervention characteristics and comparing against the planned groups for each synthesis (item #5)). | Page 9-11, Supp |
|  | 13b | Describe any methods required to prepare the data for presentation or synthesis, such as handling of missing summary statistics, or data conversions. | Page 9-11, Supp |
|  | 13c | Describe any methods used to tabulate or visually display results of individual studies and syntheses. | Page 9-11, Supp |
|  | 13d | Describe any methods used to synthesize results and provide a rationale for the choice(s). If meta-analysis was performed, describe the model(s), method(s) to identify the presence and extent of statistical heterogeneity, and software package(s) used. | Page 9-11, Supp |
|  | 13e | Describe any methods used to explore possible causes of heterogeneity among study results (e.g. subgroup analysis, meta-regression). | Page 9-11, Supp |
|  | 13f | Describe any sensitivity analyses conducted to assess robustness of the synthesized results. | Page 9-11, Supp |
| Reporting bias assessment | 14 | Describe any methods used to assess risk of bias due to missing results in a synthesis (arising from reporting biases). | Page 9-11, Supp |
| Certainty assessment | 15 | Describe any methods used to assess certainty (or confidence) in the body of evidence for an outcome. | Page 9-11, Supp |
| **RESULTS** | | |  |
| Study selection | 16a | Describe the results of the search and selection process, from the number of records identified in the search to the number of studies included in the review, ideally using a flow diagram. | Page 12 |
|  | 16b | Cite studies that might appear to meet the inclusion criteria, but which were excluded, and explain why they were excluded. | Page 12 |
| Study characteristics | 17 | Cite each included study and present its characteristics. | Page 12-13 |
| Risk of bias in studies | 18 | Present assessments of risk of bias for each included study. | Page 14 |
| Results of individual studies | 19 | For all outcomes, present, for each study: (a) summary statistics for each group (where appropriate) and (b) an effect estimate and its precision (e.g. confidence/credible interval), ideally using structured tables or plots. | Page 12-15 |
| Results of syntheses | 20a | For each synthesis, briefly summarise the characteristics and risk of bias among contributing studies. | Page 12-15 |
|  | 20b | Present results of all statistical syntheses conducted. If meta-analysis was done, present for each the summary estimate and its precision (e.g. confidence/credible interval) and measures of statistical heterogeneity. If comparing groups, describe the direction of the effect. | Page 12-15 |
|  | 20c | Present results of all investigations of possible causes of heterogeneity among study results. | Page 12-15 |
|  | 20d | Present results of all sensitivity analyses conducted to assess the robustness of the synthesized results. | Page 12-15 |
| Reporting biases | 21 | Present assessments of risk of bias due to missing results (arising from reporting biases) for each synthesis assessed. | Page 12-15 |
| Certainty of evidence | 22 | Present assessments of certainty (or confidence) in the body of evidence for each outcome assessed. | Page 12-15 |
| **DISCUSSION** | | |  |
| Discussion | 23a | Provide a general interpretation of the results in the context of other evidence. | Page 16 |
|  | 23b | Discuss any limitations of the evidence included in the review. | Page 16-18 |
|  | 23c | Discuss any limitations of the review processes used. | Page 16-18 |
|  | 23d | Discuss implications of the results for practice, policy, and future research. | Page 16-18 |
| **OTHER INFORMATION** | | |  |
| Registration and protocol | 24a | Provide registration information for the review, including register name and registration number, or state that the review was not registered. | - |
|  | 24b | Indicate where the review protocol can be accessed, or state that a protocol was not prepared. | - |
|  | 24c | Describe and explain any amendments to information provided at registration or in the protocol. | - |
| Support | 25 | Describe sources of financial or non-financial support for the review, and the role of the funders or sponsors in the review. | Page 4-5 |
| Competing interests | 26 | Declare any competing interests of review authors. | Page 4-5 |
| Availability of data, code and other materials | 27 | Report which of the following are publicly available and where they can be found: template data collection forms; data extracted from included studies; data used for all analyses; analytic code; any other materials used in the review. | Supp. |

## Supplemental table 2 : Study characteristics

| ID | Publication year | Author | Type | Countries | Centers | Nr. recruited | FU length (mean of months) [SD] | Presence of ArcticFront | Type of ArcticFront catheter used |
| --- | --- | --- | --- | --- | --- | --- | --- | --- | --- |
| 1 | 2021 | Tilz et al. | Prospective | Germany | Multicentric | 50 | NA [NA] | Yes | Cryoballoon 4th generation |
| 2 | 2021 | Anic et al. | Prospective | USA | Multicentric | 30 | 12 [NA] | No |  |
| 3 | 2021 | Creta et al. | Prospective | England | Monocentric | 80 | NA [NA] | Yes | Cryoballoon 2nd generation |
| 4 | 2021 | Kochi et al. | Prospective | Italy | Monocentric | 70 | NA [NA] | Yes | Cryoballoon 4th generation |
| 5 | 2021 | Yap et al. | Prospective | International | Multicentric | 110 | 1 [NA] | Yes | Cryoballoon 4th generation |
| 6 | 2023 | Tanese et al. | Prospective | international | Multicentric | 275 | 15 [5] | Yes | Cryoballoon 4th generation |
| 7 | 2021 | Mojica et al. | Retrospective | Belgium | Monocentric | 202 | NA [NA] | Yes | Cryoballoon 4th generation |
| 8 | 2023 | Martin et al. | Prospective | international | Multicentric | 399 | 3 [NA] | No |  |
| 9 | 2022 | Moser et al. | Prospective | Germany | Monocentric | 100 | NA [NA] | Yes | Cryoballoon 4th generation |
| 10 | 2022 | Bisignani et al. | Prospective | Belgium | Monocentric | 80 | NA [NA] | Yes | Cryoballoon 4th generation |
| 11 | 2022 | Heeger et al. | Prospective | international | Multicentric | 317 | 7.5 [3.8] | No |  |
| 12 | 2023 | Heeger et al. | Prospective | Germany | Monocentric | 205 | 12 [NA] | Yes | Cryoballoon 4th generation |
| 13 | 2023 | Honarbakhsh et al. | Prospective | United Kingdom | Multicentric | 1,688 | 30 [NA] | Yes | Cryoballoon 2nd generation |
| 14 | 2023 | Menger et al. | Prospective | Germany | Monocentric | 122 | 12 [NA] | Yes | Cryoballoon 4th generation |
| 15 | 2022 | Knecht et al. | Prospective | Switzerland | Multicentric | 80 | NA [NA] | Yes | Cryoballoon 4th generation |
| 16 | 2022 | Guckel et al. | Retrospective | Germany | Monocentric | 687 | 12 [NA] | Yes | Cryoballoon 4th generation |
| 17 | 2022 | Iacopino et al. | Prospective | Italy | Multicentric | 69 | 1 [0] | No |  |
| 18 | 2023 | Kupusovic et al. | Retrospective | Germany | Monocentric | 26 | 6 [NA] | No |  |
| 19 | 2022 | Martin et al. | Prospective | international | Multicentric | 58 | 12 [NA] | No |  |
| 20 | 2022 | Spera et al. | Prospective | Italy | Multicentric | 29 | 11.1 [2.5] | No |  |
| 21 | 2021 | Bianchi et al. | Prospective | Italy | Multicentric | 127 | 15 [5] | No |  |
| 22 | 2023 | Knappe et al. | Retrospective | Germany | Monocentric | 230 | NA [NA] | Yes | Cryoballoon 4th generation |
| 23 | 2024 | Reichlin et al. | RCT | Switzerland | Multicentric | 202 | 12 [0] | Yes | Cryoballoon 4th generation |
| 24 | 2024 | Ellenbogen et al. | Prospective | international | Multicentric | 404 | 12 [NA] | No |  |

FU = follow-up, SD = standard deviation.

## Supplemental table 3 : Patients characteristics per study and per ablation system

| ID | Main author and pub. year | Group | Nr. patients | Perc. of women | Age (mean, y (SD)) | BMI (mean kg/m2 (SD)) | LAVI (mean, ml/m2(SD)) | LA size (mean, cm(SD)) | LVEF (mean, %(SD)) | Perc. stroke | Perc. HTN | Perc DM | Perc CHF | Perc parox AF | Perc BB |
| --- | --- | --- | --- | --- | --- | --- | --- | --- | --- | --- | --- | --- | --- | --- | --- |
| 1 | Tilz et al., 2021 | POLARx | 25 | 48 % | 67.75 (2.75) |  |  | 26.25 (1.26) |  |  | 80 % | 12 % | 32 % | 48 % |  |
| 1 | Tilz et al., 2021 | ArcticFront | 25 | 32 % | 68 (4) |  |  | 29.5 (2.5) |  |  | 72 % | 12 % | 16 % | 36 % |  |
| 2 | Anic et al., 2021 | POLARx | 30 | 43.33 % | 63 (11) |  |  | 43 (4) | 63.9 (7.6) | 0 % | 53.33 % |  |  | 100 % |  |
| 3 | Creta et al., 2021 | POLARx | 40 | 35 % | 62.8 (11.7) |  |  | 38.3 (4.1) |  |  | 42.5 % | 2.5 % |  | 70 % |  |
| 3 | Creta et al., 2021 | ArcticFront | 40 | 40 % | 65 (12.1) |  |  | 40.2 (6) |  |  | 35 % | 2.5 % |  | 47.5 % |  |
| 4 | Kochi et al., 2021 | POLARx | 20 | 40 % | 62.15 (0.86) |  |  | 35.25 (2.76) | 60.75 (2.25) | 5 % | 60 % | 5 % |  | 95 % |  |
| 4 | Kochi et al., 2021 | ArcticFront | 50 | 16 % | 61.3 (4.3) |  |  | 35 (3.01) | 61.92 (2.58) | 8 % | 30 % | 6 % |  | 94 % |  |
| 5 | Yap et al., 2021 | POLARx | 57 | 42.11 % | 61.25 (2.25) |  |  | 40.5 (2) | 62.75 (1.25) |  | 31.58 % | 5.26 % |  | 75.44 % |  |
| 5 | Yap et al., 2021 | ArcticFront | 53 | 32.08 % | 63.75 (3.25) |  |  | 40.5 (1.5) | 61.25 (1.26) |  | 58.49 % | 5.66 % |  | 75.47 % |  |
| 6 | Tanese et al., 2023 | ArcticFront | 130 | 37.69 % | 63.2 (11.1) |  |  |  |  |  | 49.23 % | 6.92 % | 3.08 % | 100 % |  |
| 6 | Tanese et al., 2023 | POLARx | 137 | 40.88 % | 63.3 (10.7) |  |  |  |  |  | 41.61 % | 7.3 % | 2.92 % | 100 % |  |
| 7 | Mojica et al., 2021 | POLARx | 30 | 33.33 % | 57.47 (15.24) |  | 31.5 (8.23) |  |  |  | 33.33 % | 3.33 % | 10 % | 100 % |  |
| 7 | Mojica et al., 2021 | ArcticFront | 30 | 40 % | 53.53 (16.24) |  | 31.87 (7.31) |  |  |  | 30 % | 6.67 % | 3.33 % | 100 % |  |
| 8 | Martin et al., 2023 | POLARx | 372 | 37.1 % | 62 (11) | 28.1 (4.9) |  |  | 57 (9) | 4.3 % | 34.95 % | 8.06 % |  | 100 % | 56.18 % |
| 9 | Moser et al., 2022 | POLARx | 50 | 18 % | 65 (4.25) | 27.5 (2) |  |  | 54.5 (4.02) |  | 60 % | 20 % | 34 % | 56 % | 84 % |
| 9 | Moser et al., 2022 | ArcticFront | 50 | 38 % | 66 (5) | 27.75 (1.75) |  |  | 53.75 (3.75) |  | 74 % | 16 % | 28 % | 40 % | 82 % |
| 10 | Bisignani et al., 2022 | POLARx | 40 | 45 % | 66.6 (12.6) |  | 48.1 (9.7) |  | 52.5 (6.7) |  | 77.5 % | 30 % |  |  |  |
| 10 | Bisignani et al., 2022 | ArcticFront | 40 | 35 % | 62.8 (11.9) |  | 49 (8.3) |  | 55 (7.5) |  | 70 % | 15 % |  |  |  |
| 11 | Heeger et al., 2022 | POLARx | 317 | 42.9 % | 64 (12) | 29 (6) |  |  |  | 5.68 % | 65.3 % | 12.3 % | 16.72 % | 65.93 % |  |
| 12 | Heeger et al., 2023 | POLARx | 103 | 45.63 % | 68.7 (10.2) |  |  | 32.9 (11.4) |  |  | 73.79 % | 11.65 % |  | 51.46 % |  |
| 12 | Heeger et al., 2023 | ArcticFront | 102 | 38.24 % | 65.7 (12) |  |  | 31.7 (9.8) |  |  | 69.61 % | 10.78 % |  | 41.18 % |  |
| 13 | Honarbakhsh et al., 2023 | POLARx | 844 | 34.95 % | 61.4 (12.8) |  |  | 42.6 (4.7) |  | 3.44 % | 30.92 % | 6.64 % |  | 65.52 % | 45.02 % |
| 13 | Honarbakhsh et al., 2023 | ArcticFront | 844 | 32.46 % | 62.3 (11.1) |  |  | 42.1 (6.7) |  | 2.73 % | 33.89 % | 7.7 % |  | 71.68 % | 50.47 % |
| 14 | Menger et al., 2023 | POLARx | 61 | 37.7 % | 63.3 (11.8) | 29.5 (5) |  | 24.3 (7) | 53.3 (7.8) | 4.92 % |  | 6.56 % |  | 63.93 % | 85.25 % |
| 14 | Menger et al., 2023 | ArcticFront | 61 | 44.26 % | 64.8 (12) | 29.2 (6.3) |  | 23 (5.2) | 53.4 (5) | 3.28 % |  | 21.31 % |  | 73.77 % | 78.69 % |
| 15 | Knecht et al., 2022 | POLARx | 40 | 35 % | 65 (11) | 27 (1) | 35.5 (2.5) |  | 59.75 (2.75) |  | 50 % |  | 12.5 % | 57.5 % |  |
| 15 | Knecht et al., 2022 | ArcticFront | 40 | 35 % | 66 (9) | 26.5 (1.5) | 41 (5.5) |  | 59.75 (2.25) |  | 50 % |  | 2.5 % | 70 % |  |
| 16 | Guckel et al., 2022 | POLARx | 86 | 31.4 % | 61.3 (11.1) | 29.6 (8) | 39.1 (6.7) |  | 52.9 (3.7) |  | 56.98 % | 10.47 % |  | 58.14 % | 82.56 % |
| 16 | Guckel et al., 2022 | ArcticFront | 601 | 28.45 % | 59.2 (20.8) | 27.9 (4.6) | 39.2 (7.3) |  | 53.5 (4.8) |  | 55.24 % | 13.81 % |  | 58.4 % | 79.87 % |
| 17 | Iacopino et al., 2022 | POLARx | 69 | 33.33 % | 60.8 (11) |  |  |  | 59 (6) |  | 46.38 % |  | 0 % | 88.41 % |  |
| 18 | Kupusovic et al., 2023 | POLARx | 11 | 0 % | 65.1 (9.4) | 27.9 (4.5) | 38.7 (18.4) |  | 55.2 (5.7) | 0 % | 90.91 % | 9.09 % | 45.45 % | 36.36 % |  |
| 19 | Martin et al., 2022 | POLARx | 58 | 36.21 % | 59 (10) |  |  |  |  |  | 29.31 % | 6.9 % | 0 % | 100 % |  |
| 20 | Spera et al., 2022 | POLARx | 29 | 37.93 % | 62 (10) |  |  |  | 56 (9) |  | 44.83 % |  | 3.45 % | 100 % |  |
| 21 | Bianchi et al., 2021 | POLARx | 125 | 28.8 % | 60.3 (11) | 26.7 (5) |  |  | 58.2 (7) |  | 42.4 % |  | 2.4 % | 16 % | 39.2 % |
| 22 | Knappe et al., 2023 | ArcticFront | 114 | 37.72 % | 68 (11.1) | 27.7 (4.5) |  | 62.92 (10.03) | 58.58 (2.13) | 8.77 % | 77.19 % | 18.42 % | 10.53 % | 48.25 % | 89.47 % |
| 22 | Knappe et al., 2023 | POLARx | 114 | 37.72 % | 67.2 (10.1) | 27.2 (3.8) |  | 60.12 (9.43) | 58.85 (1.95) | 11.4 % | 80.7 % | 13.16 % | 9.65 % | 62.28 % | 80.7 % |
| 23 | Reichlin et al., 2024 | ArcticFront | 102 | 23.53 % | 62.2 (9.3) | 26.9 (4.8) | 34 (10) | 40 (7) | 61 (6) | 3.92 % | 54.9 % | 5.88 % |  | 100 % |  |
| 23 | Reichlin et al., 2024 | POLARx | 99 | 35.35 % | 62.2 (10.1) | 26.9 (4.8) | 34 (12) | 40 (6) | 60 (5) | 8.08 % | 48.48 % | 7.07 % |  | 100 % |  |
| 24 | Ellenbogen et al., 2024 | POLARx | 325 | 38.15 % | 62 (11) | 29 (6) |  | 3.8 (0.7) | 58.6 (5.8) |  |  |  |  | 100 % |  |

Characteristics are reported for POLARx and the ArcticFront Arctic Front group (when a ArcticFront system was reported by the study). AF = atrial fibrillation, BB = Beta-blockers, BMI = Body mass index, CHF = congestive heart failure, DM = Diabetes Mellitus, LA = Left atrium, LVEF = Left ventricular ejection fraction

## Supplemental table 4 : Acute efficacy outcomes per study and per ablation system

| ID | Main author and pub. year | Group | Nr of freeze per patient | Nr of CB cycles until PVI | PVI isolation per patient | PVI isolation per vein | Technical failure | Interruption of freeze due to low temperatures | Single shot isolation |
| --- | --- | --- | --- | --- | --- | --- | --- | --- | --- |
| 1 | Tilz et al., 2021 | POLARx |  | 1.1 (0.4) |  | 100 % |  |  | 68 % |
| 1 | Tilz et al., 2021 | ArcticFront |  | 1.2 (0.5) |  | 100 % |  |  | 45.36 % |
| 2 | Anic et al., 2021 | POLARx |  | 1.4 (0.4) | 100 % | 100 % |  |  | 74.17 % |
| 3 | Creta et al., 2021 | POLARx | 7.25 (2.25) |  | 95 % |  |  |  | 57.59 % |
| 3 | Creta et al., 2021 | ArcticFront | 6.25 (1.76) |  | 97.5 % |  |  |  |  |
| 4 | Kochi et al., 2021 | POLARx |  |  | 100 % |  |  |  |  |
| 4 | Kochi et al., 2021 | ArcticFront |  |  | 100 % |  |  |  |  |
| 5 | Yap et al., 2021 | POLARx | 5 (0.5) |  |  | 99.54 % |  |  |  |
| 5 | Yap et al., 2021 | ArcticFront | 5 (0.5) |  |  | 100 % |  |  |  |
| 7 | Mojica et al., 2021 | POLARx |  | 1.09 (0.3) |  |  |  |  |  |
| 7 | Mojica et al., 2021 | ArcticFront |  | 1.19 (0.5) |  |  |  |  |  |
| 8 | Martin et al., 2023 | POLARx |  | 1.5 (1.1) |  | 96.8 % |  |  | 71.24 % |
| 9 | Moser et al., 2022 | POLARx | 5 (0.5) |  |  | 99.49 % |  |  |  |
| 9 | Moser et al., 2022 | ArcticFront | 4.5 (0.25) |  |  | 100 % |  |  |  |
| 10 | Bisignani et al., 2022 | POLARx | 5 (1.3) |  | 100 % |  |  | 10 % |  |
| 10 | Bisignani et al., 2022 | ArcticFront | 5.2 (1.5) |  |  |  |  |  |  |
| 11 | Heeger et al., 2022 | POLARx |  | 1.2 (0.5) |  | 99.68 % |  |  |  |
| 12 | Heeger et al., 2023 | POLARx |  | 1.2 (0.4) |  | 99.76 % |  |  |  |
| 12 | Heeger et al., 2023 | ArcticFront |  | 1.2 (0.5) |  | 100 % |  |  |  |
| 13 | Honarbakhsh et al., 2023 | POLARx | 5.9 (2.2) | 5.9 (2.2) |  |  |  |  |  |
| 13 | Honarbakhsh et al., 2023 | ArcticFront | 6 (2) | 6 (2) |  |  |  |  |  |
| 14 | Menger et al., 2023 | POLARx |  |  |  | 100 % |  |  |  |
| 14 | Menger et al., 2023 | ArcticFront |  |  |  | 100 % |  |  |  |
| 15 | Knecht et al., 2022 | ArcticFront |  |  | 100 % | 100 % |  |  | 71.07 % |
| 15 | Knecht et al., 2022 | POLARx |  |  | 95 % | 98.73 % |  |  | 73.42 % |
| 16 | Guckel et al., 2022 | POLARx |  |  | 100 % |  |  |  |  |
| 16 | Guckel et al., 2022 | ArcticFront |  |  | 100 % |  |  |  |  |
| 17 | Iacopino et al., 2022 | POLARx | 5.3 (1.5) |  | 100 % | 63.56 % |  | 5.8 % | 77.37 % |
| 18 | Kupusovic et al., 2023 | POLARx |  | 7.6 (2) |  |  |  |  |  |
| 19 | Martin et al., 2022 | POLARx |  | 1.3 (0.7) |  | 99.57 % |  |  | 75.76 % |
| 20 | Spera et al., 2022 | POLARx |  |  |  | 100 % |  |  | 10.53 % |
| 21 | Bianchi et al., 2021 | POLARx |  |  | 100 % | 100 % |  |  | 13.23 % |
| 23 | Reichlin et al., 2024 | ArcticFront | 5.7 (1.9) |  |  | 99.24 % |  |  |  |
| 23 | Reichlin et al., 2024 | POLARx | 5.6 (2.1) |  |  | 99.22 % |  |  |  |
| 24 | Ellenbogen et al., 2024 | POLARx | 1.8 (1.32) |  |  |  |  |  | 55.89 % |

Outcomes are reported for POLARx and the ArcticFront Arctic Front group (when a ArcticFront system was reported by the study). CB = Cryoballoon, PVI = Pulmonary vein isolation

## Supplemental table 5 : Safety outcomes in the each study and group

| ID | Main author and pub. year | Complication | % in POLARx group (n/tot) | % in ArcticFront group (n/tot) |
| --- | --- | --- | --- | --- |
| 1 | Tilz et al., 2021 | Phrenic nerve palsy | 4% (1/25) | 4% (1/25) |
| 1 | Tilz et al., 2021 | Air embolism | 4% (1/25) | 0% (0/0) |
| 2 | Anic et al., 2021 | Phrenic nerve palsy | 3.3% (1/30) | 0% (0/0) |
| 3 | Creta et al., 2021 | Phrenic nerve palsy | 2.5% (1/40) | 2.5% (1/40) |
| 3 | Creta et al., 2021 | Cardiac tamponade | 2.5% (1/40) | 0% (0/0) |
| 3 | Creta et al., 2021 | Hematoma | 2.5% (1/40) | 0% (0/0) |
| 3 | Creta et al., 2021 | Hemoptysis | 0% (0/0) | 2.5% (1/40) |
| 4 | Kochi et al., 2021 | Phrenic nerve palsy | 0% (0/0) | 6% (3/50) |
| 4 | Kochi et al., 2021 | Pericardial effusion | 0% (0/0) | 2% (1/50) |
| 5 | Yap et al., 2021 | Phrenic nerve palsy | 3.5% (2/57) | 3.8% (2/53) |
| 5 | Yap et al., 2021 | Hematoma | 1.8% (1/57) | 0% (0/0) |
| 5 | Yap et al., 2021 | TIA | 1.8% (1/57) | 0% (0/0) |
| 6 | Tanese et al., 2023 | Phrenic nerve palsy | 15.3% (21/137) | 6.9% (9/130) |
| 7 | Mojica et al., 2021 | Phrenic nerve palsy | 3.3% (1/30) | 3.3% (1/30) |
| 8 | Martin et al., 2023 | Stroke | 0.3% (1/372) | 0% (0/0) |
| 8 | Martin et al., 2023 | Phrenic nerve palsy | 1.6% (6/372) | 0% (0/0) |
| 8 | Martin et al., 2023 | Air embolism | 0.5% (2/372) | 0% (0/0) |
| 8 | Martin et al., 2023 | Cardiac tamponade | 0.5% (2/372) | 0% (0/0) |
| 8 | Martin et al., 2023 | Myocardial infarction | 0.3% (1/372) | 0% (0/0) |
| 8 | Martin et al., 2023 | Gastroparesis | 0.3% (1/372) | 0% (0/0) |
| 9 | Moser et al., 2022 | Phrenic nerve palsy | 4% (2/50) | 4% (2/50) |
| 9 | Moser et al., 2022 | Thromboembolic events | 4% (2/50) | 0% (0/0) |
| 10 | Bisignani et al., 2022 | Phrenic nerve palsy | 7.5% (3/40) | 10% (4/40) |
| 10 | Bisignani et al., 2022 | Pericardial effusion | 2.5% (1/40) | 0% (0/0) |
| 11 | Heeger et al., 2022 | Stroke | 0.6% (2/317) | 0% (0/0) |
| 11 | Heeger et al., 2022 | Phrenic nerve palsy | 4.1% (13/317) | 0% (0/0) |
| 11 | Heeger et al., 2022 | Air embolism | 1.6% (5/317) | 0% (0/0) |
| 11 | Heeger et al., 2022 | Pericardial effusion | 1.6% (5/317) | 0% (0/0) |
| 11 | Heeger et al., 2022 | TIA | 0.3% (1/317) | 0% (0/0) |
| 11 | Heeger et al., 2022 | Aneurysma spurium | 0.3% (1/317) | 0% (0/0) |
| 11 | Heeger et al., 2022 | Minor bleeding | 0.6% (2/317) | 0% (0/0) |
| 12 | Heeger et al., 2023 | Stroke | 1.9% (2/103) | 0% (0/0) |
| 12 | Heeger et al., 2023 | Phrenic nerve palsy | 6.8% (7/103) | 6.9% (7/102) |
| 12 | Heeger et al., 2023 | Air embolism | 1.9% (2/103) | 0% (0/0) |
| 12 | Heeger et al., 2023 | AV block III° likely due to the intervention | 1% (1/103) | 0% (0/0) |
| 12 | Heeger et al., 2023 | Aneurysma spurium | 1% (1/103) | 2% (2/102) |
| 12 | Heeger et al., 2023 | Minor bleeding | 1% (1/103) | 0% (0/0) |
| 12 | Heeger et al., 2023 | Cardiac tamponade | 0% (0/0) | 1% (1/102) |
| 12 | Heeger et al., 2023 | Sinus arrest | 0% (0/0) | 1% (1/102) |
| 13 | Honarbakhsh et al., 2023 | Cardiac tamponade | 0.4% (3/844) | 0.4% (3/844) |
| 13 | Honarbakhsh et al., 2023 | Hematoma | 0.4% (3/844) | 0.5% (4/844) |
| 13 | Honarbakhsh et al., 2023 | Gastroparesis | 0.1% (1/844) | 0% (0/0) |
| 13 | Honarbakhsh et al., 2023 | Phrenic nerve palsy | 0% (0/0) | 0.2% (2/844) |
| 14 | Menger et al., 2023 | Phrenic nerve palsy | 4.9% (3/61) | 3.3% (2/61) |
| 15 | Knecht et al., 2022 | Stroke | 2.5% (1/40) | 0% (0/0) |
| 15 | Knecht et al., 2022 | Phrenic nerve palsy | 2.5% (1/40) | 0% (0/0) |
| 15 | Knecht et al., 2022 | Pericarditis | 5% (2/40) | 0% (0/0) |
| 16 | Guckel et al., 2022 | Cardiac tamponade | 1.2% (1/86) | 0.3% (2/601) |
| 16 | Guckel et al., 2022 | Phrenic nerve palsy | 0% (0/0) | 0.2% (1/601) |
| 16 | Guckel et al., 2022 | Hematoma | 0% (0/0) | 0.2% (1/601) |
| 17 | Iacopino et al., 2022 | Phrenic nerve palsy | 1.4% (1/69) | 0% (0/0) |
| 19 | Martin et al., 2022 | Phrenic nerve palsy | 6.9% (4/58) | 0% (0/0) |
| 19 | Martin et al., 2022 | Thromboembolic events | 1.7% (1/58) | 0% (0/0) |
| 21 | Bianchi et al., 2021 | Phrenic nerve palsy | 1.6% (2/125) | 0% (0/0) |
| 22 | Knappe et al., 2023 | Phrenic nerve palsy | 2.6% (3/114) | 1.8% (2/114) |
| 22 | Knappe et al., 2023 | Hematoma | 1.8% (2/114) | 3.5% (4/114) |
| 23 | Reichlin et al., 2024 | Phrenic nerve palsy | 5.1% (5/99) | 0% (0/0) |
| 24 | Ellenbogen et al., 2024 | Phrenic nerve palsy | 1.2% (4/325) | 0% (0/0) |
| 24 | Ellenbogen et al., 2024 | Air embolism | 0.3% (1/325) | 0% (0/0) |
| 24 | Ellenbogen et al., 2024 | Cardiac tamponade | 0.6% (2/325) | 0% (0/0) |
| 24 | Ellenbogen et al., 2024 | Hematoma | 1.5% (5/325) | 0% (0/0) |
| 24 | Ellenbogen et al., 2024 | Myocardial infarction | 0.3% (1/325) | 0% (0/0) |
| 24 | Ellenbogen et al., 2024 | Gastroparesis | 0.9% (3/325) | 0% (0/0) |
| 24 | Ellenbogen et al., 2024 | Pulmonary edema | 0.3% (1/325) | 0% (0/0) |

Characteristics are reported for POLARx and the ArcticFront Arctic Front group (when a ArcticFront system was reported by the study). AV = Atrioventricular, TIA = Transient ischemic attack. N = number of patient with the complication, tot = total number of patient in the cohort/subgroup

## Supplemental table 6 : Latest timepoint the persistent PNP were documented

| Latest time point the persistent PNP was documented | POLARx | ArcticFront |
| --- | --- | --- |
| Persistent at least on the first post-intervention day | 1 | 1 |
| Persistent at discharge | 7 | 6 |
| Resolved at 1 month | 1 | 0 |
| Unresolved at 1 month | 0 | 1 |
| Resolved at 3 months | 1 | 0 |
| Unresolved at 3 months | 4 | 0 |
| Unresolved at 6 months | 7 | 0 |
| Resolved at 12 months | 6 | 3 |
| Unresolved at 12 months | 1 | 1 |

## Supplemental table 7 : Procedural details per study and per ablation system

| ID | Main author and pub. year | Group | Nadir temperature | Temperature at isolation | Time to isolation | Procedural time | Fluoroscopy time | Ablation time |
| --- | --- | --- | --- | --- | --- | --- | --- | --- |
| 1 | Tilz et al., 2021 | POLARx | -57 (7) |  | 48 (32) | 45.5 (3.5) | 8.5 (1.5) |  |
| 1 | Tilz et al., 2021 | ArcticFront | -50 (6) |  | 41 (23) | 55 (2.5) | 12 (1.5) |  |
| 2 | Anic et al., 2021 | POLARx | -53.1 (5.3) |  | 59 (40.7) | 135 (30) | 13.4 (8.5) |  |
| 3 | Creta et al., 2021 | POLARx | -56.9 (0.8) |  | 17.25 (4.75) | 81 (29.16) |  |  |
| 3 | Creta et al., 2021 | ArcticFront | -48.6 (1) |  | 16 (6.5) | 77.5 (32.6) |  |  |
| 4 | Kochi et al., 2021 | POLARx | -57.45 (1.15) | -43.5 (3.5) | 45.5 (9.1) | 90 (15) | 15.78 (2.33) | 16.05 (1.81) |
| 4 | Kochi et al., 2021 | ArcticFront | -47.48 (1.28) | -32.23 (2.08) | 39.65 (5.65) | 60.5 (5.5) | 13.92 (1.78) | 14.75 (0.75) |
| 5 | Yap et al., 2021 | POLARx | -55.25 (2.25) |  | 48 (9.01) | 81.75 (6.25) | 14.03 (2.13) |  |
| 5 | Yap et al., 2021 | ArcticFront | -47.25 (2.25) |  | 45.5 (9.01) | 66.5 (8.5) | 11.45 (2) |  |
| 6 | Tanese et al., 2023 | POLARx |  |  |  | 74.8 (24) | 14.8 (8.4) |  |
| 6 | Tanese et al., 2023 | ArcticFront |  |  |  | 78.8 (25.4) | 16.5 (8.3) |  |
| 7 | Mojica et al., 2021 | POLARx | -58.13 (6.26) |  |  | 60.5 (14.23) | 12.83 (6.03) |  |
| 7 | Mojica et al., 2021 | ArcticFront | -49.63 (6.19) |  |  | 73.43 (13.26) | 17.23 (7.17) |  |
| 8 | Martin et al., 2023 | POLARx | -56.6 (6.5) |  | 50.2 (34.1) | 68.2 (24.6) | 15.6 (9.6) |  |
| 9 | Moser et al., 2022 | POLARx | -60 (2.5) | -40.75 (5.27) | 36.25 (5.25) | 81.25 (11.25) | 17.25 (2.25) |  |
| 9 | Moser et al., 2022 | ArcticFront | -48.75 (2.25) | -30.25 (4.25) | 31 (5.5) | 61.56 (9.44) | 11.25 (2.25) |  |
| 10 | Bisignani et al., 2022 | POLARx | -59.9 (4.02) | -38.4 (11.86) | 37.7 (9) | 107.9 (51.2) | 29.4 (14) |  |
| 10 | Bisignani et al., 2022 | ArcticFront | -49.2 (5.2) | -32.6 (4.98) | 34.1 (11.7) | 101.59 (32.5) | 24.7 (8) |  |
| 11 | Heeger et al., 2022 | POLARx |  |  | 46.1 (28.5) | 92 (41) | 15 (10) |  |
| 12 | Heeger et al., 2023 | POLARx | -56.1 (8.3) |  | 42.4 (27.7) | 54.5 (17.1) | 9.3 (4.3) |  |
| 12 | Heeger et al., 2023 | ArcticFront | -46.9 (10.1) |  | 42.2 (28.3) | 59.4 (18.6) | 12.5 (9.3) |  |
| 13 | Honarbakhsh et al., 2023 | POLARx |  |  |  | 78.6 (38.1) | 16.1 (12.3) |  |
| 13 | Honarbakhsh et al., 2023 | ArcticFront |  |  |  | 79.4 (25.8) | 16.7 (11.4) |  |
| 14 | Menger et al., 2023 | POLARx |  |  |  | 37.8 (12.7) | 12.1 (6.8) |  |
| 14 | Menger et al., 2023 | ArcticFront |  |  |  | 37.9 (9.2) | 13 (7.7) |  |
| 16 | Guckel et al., 2022 | POLARx |  |  |  | 113.9 (23.4) | 10.9 (7.1) |  |
| 16 | Guckel et al., 2022 | ArcticFront |  |  |  | 100.7 (32.5) | 8.4 (7.5) |  |
| 17 | Iacopino et al., 2022 | POLARx | -56.25 (2.25) |  | 46.75 (9.26) |  |  | 195 (15.16) |
| 18 | Kupusovic et al., 2023 | POLARx |  |  |  | 177.3 (56.5) | 23.1 (7) |  |
| 19 | Martin et al., 2022 | POLARx |  |  |  | 107.6 (26.1) | 14.1 (9) | 17 (5.4) |
| 20 | Spera et al., 2022 | POLARx | -55.75 (1.75) | -42.75 (2.25) | 38.5 (4.5) |  | 18.6 (8) | 17.9 (5) |
| 21 | Bianchi et al., 2021 | POLARx | -56.75 (2.25) | -47 (3) | 42.25 (7.76) |  |  |  |
| 22 | Knappe et al., 2023 | POLARx |  |  |  | 71.25 (8.75) | 11.25 (1.55) |  |
| 22 | Knappe et al., 2023 | ArcticFront |  |  |  | 69.5 (8) | 10.6 (1.5) |  |
| 23 | Reichlin et al., 2024 | ArcticFront |  |  |  | 68.1 (24.8) | 15.7 (7.9) |  |
| 23 | Reichlin et al., 2024 | POLARx |  |  |  | 73.1 (27.3) | 15.4 (7.4) |  |
| 24 | Ellenbogen et al., 2024 | POLARx | -56.43 (6.85) |  | 45.11 (25.28) |  |  | 4.46 (2.68) |

Characteristics are reported for POLARx and the ArcticFront Arctic Front group (when a ArcticFront system was reported by the study).

# Supplemental figures

## Supplemental figure 1 : Flow chart

## Supplemental Figure 2 : Acute efficacy outcome

**A)**

**B)**

Weighted proportion of acute efficacy outcome among all studies reporting data for the POLARx ablation system. The number of events represent the number of successful ablation (A) per patient, B) per vein). CI = Confidence interval

## Supplemental Figure 3 : Weighted percentage of phrenic nerve palsies using POLARx accross all studies reporting this endpoint

Weighted proportion of phrenic nerve palsies (PNP) among all studies reporting data for the POLARx ablation system. The number of events represent the number of PNP. CI = Confidence interval

## Supplemental figure 4: Procedural time outcomes

**A)**

**B)**

**C)**

Weighted mean procedural time, fluoroscopy time and time to isolation among all studies reporting data for the POLARx ablation system. WMD = Weighted Mean Difference, SE = Standard Error, CI = Confidence interval

## Supplemental Figure 5d : Procedural temperature outcomes

**A)**

**B)**

Weighted mean of temperature at isolation and nadir temperature (both in degree Celsius) among all studies reporting data for the POLARx ablation system. WMD = Weighted Mean Difference, SE = Standard Error, CI = Confidence interval

## Supplemental Figure 6 : Studies quality

Study quality assessed using the Newcastle-Ottawa scale (NOS) for observational studies and the ROBINS-I tool for randomized controlled trials.
